# ‘Everyone is trying to outcompete each other’: a qualitative study of medical student attitudes to a novel peer‐assessed undergraduate teamwork module

**DOI:** 10.1002/2211-5463.13395

**Published:** 2022-03-23

**Authors:** Helen R. Watson, Mary‐Kate Dolley, Mohammad Perwaiz, Jocelyn Saxelby, Gianluca Bertone, Steven Burr, Tracey Collett, Robert Jeffery, Daniel Zahra

**Affiliations:** ^1^ 6633 Peninsula Medical School University of Plymouth UK; ^2^ 6633 Peninsula Dental School University of Plymouth UK; ^3^ 6634 University Hospitals Plymouth NHS Trust Plymouth UK

**Keywords:** employability, qualitative study, skill assessment, students as partners, teamwork, transferable skills

## Abstract

The centrality of teamwork in ensuring the effective functioning of institutions across all sectors is undeniable. However, embedding teamwork into higher education has been hampered due to a range of deeply entrenched practices associated broadly with the foregrounding of knowledge, beliefs about the place of skill training and routines of assessment. As a result, despite an urgent need to address teamwork, little progress has been made with respect to progressing teamwork education. We have designed and evaluated a novel teamwork module delivered to fourth‐year undergraduate medical students involving placements, a cocreated piece of work, reflection and summative peer assessment. This paper aimed to investigate whether the module increased students’ insight into teamwork, including their own skill development, and whether their perceptions of teamwork changed. Throughout the evaluation, students played a key role, with four final‐year medical students working alongside others in the multidisciplinary project team. Five distinct themes emerged from our in‐depth, semi‐structured interviews: (a) importance and meaning; (b) insight into skill development; (c) transferability; (d) peer assessment; and (e) resistance to teamwork education. Themes had positive and negative components, and student perceptions changed in multiple ways after experiencing a longitudinal educational opportunity to develop their teamwork skills. Before practice, students focused on superficial explanations and on where they might improve. In contrast, after practice, students conveyed deeper insights, contextualisation, focus on how they might improve, and shared structured reflection.

## Graphical abstract

This article explores medical students’ perspectives on teamwork, in the context of a peer‐assessed teamwork module. We discuss our attempts to develop and assess teamwork in UK undergraduates and consider the challenges of teamwork education and assessment. Here, we present a detailed qualitative analysis of our students’ experiences and discuss good practice in teaching this crucial transferable skill.

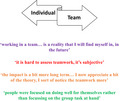

**Abbreviations**
BMBS: Bachelor of Medicine Bachelor of Surgery
GMC: General Medical Council
HE: higher education
PMS: Peninsula Medical School
SSC: student selected component
SSU: student selected unit
WHO: World Health Organisation

It is almost impossible to think of an area of work or society in which the ability to work in a team is not important. To be ready for the workplace, graduates must be equipped with vital transferable skills and be able to articulate them with insight and specific examples [1, 2].

This study offers an in‐depth exploration of UK medical undergraduate students’ perceptions of teamwork and teamwork education, in relation to a novel teamwork module. This study aimed to explore whether students had any greater insight into teamwork, in terms of the concepts of teamwork and their own skill development, as a result of the module and whether their perceptions of teamwork had changed. Whilst this study was carried out in a UK‐based medical training context, the findings and questions raised are very relevant across higher education (HE) internationally, including for those involved in delivering and developing molecular bioscience education.

Teamwork has been defined in a number of ways, but often, these definitions include some reference to the size of group (two or more), having common goals and some benefit or synergy arising from team members working together [3]. Depending on the context, definitions of teamwork may focus on the end product the team produces, the individual contributions of team members and processes adopted by the team, or, in the case of our approach, a combination of both [4, 5, 6]. In order to work successfully as a team, several behaviours and attitudes are required of team members, and these factors can be used in an educational context to ‘measure’ teamwork ability. Some of these are reflected in our peer feedback criteria for this module (respect for others’ roles, working constructively, communication and being supportive and fair). Some examples of other attitudes and behaviours important to teamwork include (but are not limited to) managing conflict, working with people from different backgrounds, time management, planning, giving feedback and leadership [6]. Therefore, being skilled at teamwork is not just one thing; rather, it is a collection of skills, behaviours and attitudes that different individuals will possess in different quantities. It is important to note that many of the attributes of a successful team member will be advantageous outside of the team setting, again reinforcing the importance of teamwork education in HE and beyond.

## Teamwork in health care and science

Medical doctors need good scientific understanding to make diagnoses and manage their patients. Teamwork and other transferable skills have also been recognised by the General Medical Council (GMC) and the World Health Organisation (WHO) as being crucial to good patient outcomes and patient safety [7, 8]. The GMC classifies complaints received into four domains: communication, partnership and teamwork; knowledge and performance; maintaining trust; and safety and quality. In 2018, communication, partnership and teamwork accounted for 1253 (20%) allegations and 69 (11%) cases proven at a hearing, compared with 3367 (52%) and 232 (36%) respectively for knowledge and performance. However, failure to get a second opinion, not consulting colleagues and not respecting the patient’s views were classified under knowledge and performance rather than a failure of teamwork [9]. Inadequate teamwork therefore accounts for a significant proportion of complaints made against doctors.

Effective multidisciplinary teams are critical to the success of healthcare systems. For example, the COVID‐19 pandemic has demonstrated that collaboration, communication and teamwork are key to managing quickly evolving situations [10, 11]. In addition, aptitude in teamwork has been shown to be a predictor of ability in clinical reasoning [12] and can enhance the retention of medical knowledge [13].

Transferable skills, including teamwork, are just as essential in other subjects and fields of work, and as educators, we should share good practice in transferable skill education across disciplines. For example, graduates of bioscience programmes must be able to work in multidisciplinary teams, to be critical and open to new information and to reflect upon and develop their skills [1]. More broadly than that, it is clear that HE across Europe needs to do more to equip graduates with skills they need to thrive in modern workplaces in all sectors [14, 15].

## Provision of teamwork education in HE

Despite the importance of teamwork, training and assessment of this skill in HE is often inadequate. Much of European education is knowledge‐based, with examinations from school onwards testing retention of facts rather than skills and problem‐solving. This is partly historical, but it may also be because testing facts is more straightforward than testing transferable skills [4, 16, 17]. In addition, some educators assume that teamwork and other skills are acquired automatically through group work. Although group work can facilitate the development of teamwork skills, specific opportunities to develop such skills are also required, and students need focused feedback to improve. In both medical and dental education, students tend to focus on content recall rather than skill development, so specific sessions and support are required to help them develop transferable skills [18, 19]. A review of UK medical graduates’ preparedness for practice strongly suggests that whilst they are competent at history taking and other clinical skills, they are unprepared to work in multidisciplinary teams [20]. This is not limited to medical education, as nursing and other healthcare professions students also demonstrate relatively poor teamwork skills compared with other competencies related to patient safety [21]. Some students also agree that current teamwork training and assessment is insufficient to prepare doctors for practice and that curricula should be revised accordingly [16, 22, 23].

## Students’ perceptions of teamwork

When developing teamwork education, it is vital to understand that students’ perspectives sometimes differ from those of their teachers; qualified doctors rate teamwork as a more important competency than their students [24]. Others have reported that fourth‐year medical students rate teamwork highly in improving patient safety, but feel they need more training to be confident [25]. Another study comparing trainees and staff at 10 American hospitals showed that trainees and staff rated ‘teamwork across units’ as similarly important for patient safety. Trainees rated ‘teamwork within units’ as more important than senior staff did [26]. Students (especially those who have had more extensive clinical exposure) appreciate the importance of teamwork opportunities and identify leadership and group management skills as key to their success, but indicate that they do not have sufficient training in these areas [27].

## Barriers to teamwork education

Teaching and assessing teamwork is challenging and complex. In medical education, the competition between students for rankings and foundation places is fierce. Competition and individual success also underpin access to HE for students in all fields. This can create dissonance as we ask students to succeed as individuals, and contribute their time and energy to team goals that may benefit their competitors [22]. Improvements in teamwork education may also be inhibited by a lack of urgency and action on the educators’ behalf, possibly related to a lack of expertise amongst educators and institutions in delivering this type of education and inadequate support from programme accreditors [28]. Assessment of teamwork is challenging, but without assessing these skills, we have no indication of whether our students are competent, or our curricula adequate. Some tools to assess teamwork have been developed, but there is still a need for more robust, authentic and fair assessment of teamwork skills in HE, not just the products of teamwork [4, 16].

## Good practice in teamwork education

The effectiveness of teamwork education can be increased by making it authentic and relevant to the students and their future careers [17, 29, 30]. Placements can demonstrate to the students how essential these skills are in the real world, and reflecting on these experiences further reinforces learning and helps students identify areas they need to develop [1, 31, 32]. Indeed, the authenticity of the environment in which transferable skills are developed is important across disciplines, for example optimising the use of fieldwork to allow students to self‐evaluate group work in geography and the value of laboratory sessions and informal study groups for science students to develop teamwork skills [33, 34]. Students’ drive to develop their own teamwork skills may be enhanced by observing and identifying positive, exemplary teamwork behaviour whilst on placement and diminished if they observe only poor teamwork [35]. Peer feedback and assessment is often part of teamwork education in HE, but developing tools that allow reliable and objective assessment by students is challenging. Ensuring students understand the rubric being used, are comfortable giving honest feedback and that they receive training in giving effective feedback all contribute to successful peer feedback experiences as demonstrated, for example, in medical education and midwifery training [36, 37, 38].

## A novel teamwork module

The student selected component (SSC) is deemed by the GMC to be an essential part of UK medical training, giving students some degree of choice of their programme content. We considered this the best place in the programme to improve our provision of teamwork education. At Peninsula Medical School (PMS), students undertake a SSC module in each of the 5 years of our Bachelor of Medicine Bachelor of Surgery (BMBS) programme, allowing longitudinal development of skills. Our SSC modules have historically focused on developing transferable skills, but emphasis had drifted towards specialist content knowledge, reducing the space for skills. This, coupled with the heavy focus on transferable skills in the GMC’s ‘Outcomes for Graduates’ [7], led us to redevelop the SSC module across all 5 years of the medical curriculum. Student selected units (SSUs) are the constituent parts of the SSC module, delivered in each year. The new SSU in year 4 of the BMBS programme was specifically designed to promote the development and assessment of teamwork. We have developed a novel, summatively assessed, teamwork module that involves placements, creation of a shared piece of work, reflection and peer feedback.

Fourth‐year students are randomly assigned to groups of eight to 10 students with a trained facilitator. They study teamwork during four 1‐week periods spread through the year, with some longitudinal work in between. The first assessment is an individual reflection of their strengths and weaknesses in teamwork and those of their teammates, and a personal development plan (Table 1). This is informed by peer feedback offered by and to each student during the 4 weeks (Table 2). The second assessment is a single, collaborative report submitted by the group (Table 3), for which each student receives the same score, modified to a small extent by a peer assessment factor.

**Table 1:** Assessment domains for the individual report.

1. Self‐appraisal of your own teamworking skills
2. Plans for personal development of your teamworking skills
3. Critical reference to evidence and literature on teamworking
4. Style, fluency and impact

**Table 2:** Assessment domains for the peer feedback.

1. Respect for others’ roles
2. Working constructively in the team
3. Communicating well
4. Being supportive and fair

**Table 3:** Assessment domains for the group report.

1. The patient’s (or their family’s) experience and involvement in their own care
2. Critical reflection on the teamwork of professionals involved in the patient’s care
3. Description and appraisal of well‐being provision in the observed teams for the patient and staff
4. Appraisal of the students’ SSU team management
5. Style, fluency and impact

To increase authenticity, each group is allocated a ‘virtual patient’ with complex medical and social needs, typical of patients seen in primary care. They are encouraged to look at the effectiveness and convenience of healthcare provision from the patient’s point of view and reflect on examples of effective and less effective teamwork they have witnessed. Students can draw on previous experiences and other parts of the curriculum, as well as spending time observing real healthcare teams in the hospital, attending multidisciplinary and management meetings, clinical events and speaking with patients. Finally, they examine their team’s experiences working together to produce the joint report.

Each student provides numerical summative peer assessment scores for other students in their team. This is a score of two, three or four, justified by the peer feedback during the year. Students are asked to give at least one, and not more than half their team, the highest score of four. The method was designed to ensure that they could not give equal marks to everyone and that each person's judgement had equal weight.

Each student’s final score for the SSU was a combination of the score for the team‐created report and ratings of their teamwork from their peers. Peer scores were adjusted to account for group size, ensuring each score carried comparable weight for students across different‐sized groups, and then, the average of these peer scores was used to adjust the team report score for each individual. The adjustment and combination of peer scores resulted in a multiplication factor of less than one for students rated as contributing below the team average, giving them an individual score below the team report score (i.e. the team score reduced for their below‐average teamworking). Conversely, the resultant multiplication factor was above one for those rated as contributing above the team average, giving an individual score above the team report score (i.e. the team score increased for their above‐average teamworking).

To evaluate our new teamwork SSU, we used semi‐structured interviews to explore our students’ perspectives and experiences and to furnish the literature with much needed qualitative evidence on teamwork education [39]. Four final‐year students were involved in the project, as equal members of the research group. They carried out interviews and participated fully in the research process alongside the other team members.

## Materials and methods

Our qualitative study was undertaken in the 2020/21 academic year, following a pilot study with three fourth‐year students in 2019/20. As our module was new, we wanted to undertake a thorough evaluation to explore our students’ perceptions of teamwork. We conducted two in‐depth interviews with each of nine fourth‐year undergraduate medical students, one before the module commenced, and the second after the final sessions had taken place (but before the group report marks were released). Our research team comprised five academic staff and four final‐year medical students. Following training in qualitative interviewing, those undertaking the interviews utilised a topic guide, included in Appendix S1. All interviewers met to agree their approach to the interviews before they interviewed their students. We agreed on a semi‐structured approach, allowing interviewers to follow up interesting points in more depth. We intended the interviews to be fairly conversational in nature. The topic guide ensured that all of the key questions were asked, but we gave interviewers the freedom to prompt and explore themes more deeply as appropriate.

This project was part of a routine evaluation, involving staff and students as investigators. The study was formally advertised to all year 4 students by staff during an induction session. More informally, student and staff members of the research team further promoted the opportunity, for example by mentioning it during teaching sessions and by student investigators sharing it with their networks. Nine students agreed to take part. Because contribution to this study was voluntary, it is possible that the students who came forward may not be representative of the full cohort. They may have had a particular interest in teamwork, or perhaps stronger feelings about the strengths or weaknesses of the module than their peers. All participants were informed of the aim of the study, that their interviews would be anonymised following data collection and that their identities would only be known to the individual interviewers. To minimise bias, we made it clear that student marks would not be impacted by participating, and emphasised the value of sharing information to benefit future educational practice. At the data analysis stage, it became clear that the findings may be helpful to the wider HE community. Although routine course and module evaluation does not require ethical approval at our institution, we wrote to all participants asking for consent to use their data for publication, and they all consented. Therefore, although retrospective to data collection, all participants have given written informed consent. Our final report was shared with participating students prior to submission for publication.

The interviews took place online (on Zoom) and lasted approximately 40 min each. Each interviewer transcribed and anonymised their interviews verbatim. The anonymised transcripts were then uploaded to a password‐protected database accessible by only the project team.

The interviews were analysed thematically by the research team. Although each interview was only transcribed once, we cross‐checked the themes arising from the transcribed interviews at the thematic analysis stage. Initially, we analysed two ‘before‐and‐after’ transcripts each. The team then met and negotiated an analytical/thematic framework based on all of the interviews. All members of the research team contributed to the thematic analysis. The data were coded to the framework by several members of the project team, using the qualitative analysis software ‘nvivo’.

Our methodological framework, that is our assumptions regarding the nature of being and knowledge fit within, what in sociological terms is referred to as, is an interpretivist paradigm (see, e.g., O'Donoghue, 2018 [40]). Aligning with social constructionist theories of learning [41], interpretivism holds that our experiences of reality are subjective, and that in research terms, the closest we can come to ‘knowing about reality’ is through accessing the accounts of others in ways that minimise interference with their versions of the truth. Whilst we position ourselves within the interpretivist tradition, in conducting this research we are not at this stage applying any one particular theoretical perspective (e.g. ‘teamwork through the lens of Bourdieu’s 1984 work on cultural capital’ [42]). Our research is exploratory with the intention of, in the first instance, describing participants’ responses in as much depth as possible.

## Results

### Importance and meaning of teamwork to students

Before the module, most students agreed that teamwork was important, particularly in the context of health care. When asked *‘How important is teamwork to you? Why?’* most answers were fairly similar, strongly agreeing that teamwork was important and sometimes also linking good teamwork to better patient care.

‘I think teamwork is an extremely important and significant skill… there are other things… especially in health care, which [require] different people working together’.

‘you’d see doctors nurses, different types of doctors, you’d have radiologists… histopathologists, all in one room… each of them would have a point and then they would discuss it through and see how to give the patient the best care possible’.

Although students initially answered in terms of health care, some also included examples of teamwork from outside of their studies.

‘cricket is a sport which is very teamwork oriented… even if you do your best and some of your other team members aren't cooperating and you don't work as a team, you don't achieve the results you want to achieve. So for me personally, I think it's a very significant skill’.

After the module, their answers to this question were very similar, but with increased insight and depth, drawing on examples from experiences and observations during the module.

‘they were talking about obviously encountering… a really poorly child and how that can… effect you emotionally quite badly as well and how you’ve got these team debriefs, you’ve got other members of the team that you can talk to… when you’re within a team… I guess you never really feel alone’.

‘teamwork is really important; it’s something I have realised throughout this year on placements and especially during this SSU. I’ve realised what makes teams work well and what doesn’t and how the team dynamic changes based on the type of people you are working with’.

Students were asked what they thought made good teamwork before and after the module. Before the module, good communication, sharing of workload and responsibility, roles in teams (including leadership), and having a common goal were mentioned.

‘when I think about teamwork, the aspects I think about that are important are definitely communication skills… I feel like the bad teams literally just don’t understand each other; they don’t try to understand each other’.

‘sharing the workload… if you can’t do something, you can ask your friends or your colleagues to do it and then in exchange you know you do something else’.

‘everyone having their role and like and kind of taking their responsibility and being trustworthy’.

‘you all need to be reasonably like‐minded as far as like you have got to kind of want the same outcome… but not necessarily have the same skill set I guess like a variety of skill sets in the team in the individual people will make it better’.

After the module, the key ideas were similar, with the addition of references to using individuals’ strengths.

‘if you can identify the strengths and weaknesses of individual team members as early as possible, then you can allocate specific tasks or ask them to take up certain roles which they would be good at, and that means you can make the most of each team member and also you know what each individual may need support with because you’ve identified their weaknesses’.

‘having a shared aim… having different types of personalities, having different members within a team as well is quite important if you have different aptitudes… if we’re all a similar personality that can be quite difficult… that’s one of the things I noticed whilst working in teams’.

We asked students about barriers to teamwork. Before the module, students generally identified the inverse of the previous question, most often mentioning poor communication and lack of a shared goal. Lack of leadership, motivation and commitment were also highlighted, as were conflict and stressful environments.

‘if you have bad communication, whether someone's being too loud or they're trying to sort of outshine people, especially sort of if you look at medical school where you're being competitive’.

‘if you don't buy into the team and if you don't feel motivated, if you don't feel… valued enough, then these are all barriers to you contributing towards the team and the team working on a team level… if no one takes responsibility, no one's in a leadership role, even if that leadership role isn't being taken up by a single individual, as long as someone's leading different phases of a project’.

‘when people aren’t committed… If one person’s not committed it kind of puts the whole team out’.

‘if people are stressed then they’re less likely to help other people… or maybe less willing to help other people, or maybe more people are fixated on completing their own set of tasks’.

After the module, some students incorporated examples from their own teamwork experiences.

‘for different parts of the SSU my team were very, kind of, unsure of what the end goal was, and I felt that we didn’t have very clear communication from our facilitator at some points… So everyone was a bit confused on what the right thing to do was’.

### Student insight into skill development

We asked students what aspects of teamwork they thought they were good at, and what they could be better at. In the first interviews, students were able to identify strengths and weaknesses, including areas they needed to work on, but there was not much discussion of how they might develop these areas.

‘completing team tasks in a timely manner… I understand the consequences of completing tasks a bit later than they should be… the whole team falling behind is very unfair’.

‘I could probably take on a lot more sometimes… I guess maybe sometimes I might take on a little bit less than that I know I can take on. But then I would say that's partially a good thing because it means I don’t kind of get overworked’.

‘I guess doing a bit more critical thinking in teams, I would like to question a bit more and try to challenge and push teams and myself a bit more’.

‘the sort of fine balance between being confident and over‐bearing… I think at the moment I’m… not quite as confident as I could be in teams’.

In the second interviews, students reflected more, showing some insight into and ownership of their own development, often drawing on experiences from this module. Some students also talked about what they might develop in future teamwork opportunities.

‘now in the future, I might try and identify certain team members and certain team roles that they've taken and be mindful of their weaknesses and if I can support them in any way, I'll be more able to do so because I have a better understanding of it’.

‘I think that it’s very important to reflect on teamwork skills, and I think it’s important to reflect upon yourself alone and also in the context of a team’.

‘I would say it probably hasn’t made a massive change since the SSU, but then again, like, it might be that the impact is a bit more long term, in that I now appreciate a bit of the theory, I sort of notice the teamwork more… I think I understand a bit more about my weakness as well, that’s certainly, that’s certainly come out this, actually’.

‘when I started off, I was quite a different leader… than I am now. I think what works best is sort of just really empowering the other members of the team… I think it’s really important to tell how everyone’s role is really really important and I think that encourages people to do just as well… I think its called the collaborative leadership or something… where you… engage the other members and empower the other members to work equally hard and just obviously give them the praise and the reward for all their hard work so I think that works the best for me personally’.

### Transferability of teamwork

Key to student development and awareness of teamwork is understanding how it transfers across different contexts. In the first interviews, students seemed well aware of the importance of teamwork in health care, and mentioned teamwork in sports and home life, though comments were generally superficial and impersonal.

‘in medicine, where you come across so many different dynamics of teams and individuals, different sort of professionals and different jobs, just working together… [if] the team sort of is not working as efficiently as it should. Then patient care will deteriorate and you'll have issues regarding patient safety’.

‘like five‐a‐side football, whoever scores the most overall you’re clearly a better team’.

In the second interviews, students talked about the transferability of teamwork with more fluency. They mentioned examples of using teamwork in various situations, and a more detailed, personal view of the importance of teamwork and similarities and subtle differences between contexts.

‘it has made me appreciate that working in a team with people you may not necessarily know previously is a reality that I will find myself in, in the future’.

‘I think you probably can do it without teamwork but it is not going to be as good for the patient, you’re probably not going to enjoy it – it’s going to be much more isolating, and you are going to have a lot more work to do yourself’.

‘the comparison I was drawing was, like in a trauma environment everything I’ve been seen has been done in a polite sort of way, but quite direct, without any sort of fluffiness on the side. But… I go on the hockey field for example if, if you’ve botched up and your teammate goes “oh come on” or something like that… I think you just get over it, I think like I don’t tend to ruminate about it… if someone says “Well, for God’s sake…,” … at you, and you’d just go well I got something wrong, but then you’d just crack on, but I personally wouldn’t reflect on that and say oh in that situation they were a bit kind of like blunt with me; therefore I’m offended, it would just be like the situation that’s the fault’.

### Peer assessment in teamwork

Students were asked explicitly in the second interview *‘What do you think about being assessed by your peers?’* but it was also raised in the first interviews by most students.

Students’ views on peer marking were mixed. Whilst they broadly acknowledged the potential positives of the approach, they were not very supportive of being assessed by their peers themselves.

First interview: ‘I think that there are some benefits, I think, it probably will make you feel, makes you more motivated to be a good team worker’.

First interview: ‘I think you should just leave, leave [assessment] to whoever's facilitating, I think that might be fairer, so then it's not like a popularity thing’.

Second interview: ‘it’s a useful and necessary thing to peer assess, and I think at our medical school it’s something that isn’t new to us as we’ve always done that since PBL and small groups from year one’.

They also identified potential problems, such as difficulty in assessing their friends, competition and whether or not they had the right skills to carry out an objective assessment of another student.

Second interview: ‘I'm sure… if people in the same friendship group, everyone's going to try and help each other go up the rankings in whatever way they can… it would be quite unfair for people who do really work hard’.

Second interview: ‘being assessed by peers didn’t worry me too much I think it was a bit harsh though because we weren’t really in a position to objectively do it’.

There was also an indication that students might artificially exhibit certain behaviours to meet assessment criteria.

Second interview: ‘you can tell that people were doing things just because they knew they were going to get marked on it. So it was very fake because – you wouldn’t do that but you know you are being marked by us all on it… You act slightly differently… it was barn door obvious when people were faking things… you know in real life that is not how they act in the team’.

### Student resistance to teamwork education

Most investigators agreed that their interviewees expressed some degree of resistance to teamwork education. Some students that felt that they had already ‘done’ teamwork, but appreciated the opportunity to exercise these skills.

Second interview: ‘I think from what we observed throughout uni before this point and from my experience in life – I feel like I have already picked up how to work as a team in a relatively professional manner’.

Second interview: ‘I feel like it has in terms of learning more about practical ways to implement more teamworking skills because I’ve always known what good teamworking skills and used them in jobs and stuff, so I think it was nice to practise implementing them in a more medical situation’.

Second interview: ‘I guess people just assume that they’re okay at work in a team anyway. .. cos I don’t think many people would think I’m rubbish at team work because you’d probably a bit of a loner sort of thing you know’.

Some students lacked faith in the module design and its ability to assess teamwork fully.

First interview: ‘it is hard to assess teamwork, it’s subjective – yeah, you can reach the endpoint – and not really have good teamwork getting there. Equally you cannot get to the end point but you can have really good teamwork’.

First interview: ‘I definitely think it’s more about what the group produces as opposed to like how effective the group has been’.

Anxiety may have caused resistance for some students, given the new and unfamiliar activities and assessments involved.

Second interview: ‘I think I was quite anxious at the start of what they were expecting from this team module’.

Some students were doubtful of whether they could really learn teamwork skills during this module, as it may be too superficial or artificial.

First interview: ‘it’s kind of hard when you make it an artificial activity – because everyone knows it is artificial and they are not really invested in the goal in the same way’.

Second interview: ‘I think that my team and a lot of other teams felt, is we were kind of going to observe teams on placement with the aim of just observing how they are. And that was a little bit difficult to communicate to them, they would be like “oh, what are you hoping to gain from today” and we were like “oh we’re just here to watch you do your meeting.” And I think that’s a little bit superficial’.

Competition between students for grades and rankings is likely to make them resistant to some degree to contribute for the good of the team.

Second interview: ‘people were focused on doing well for themselves rather than focussing on the group task at hand. I think that’s understandable because your decile rank is going to be affected badly if you don’t do well and everyone is trying to outcompete each other’.

Second interview: ‘The primary thing in all group members’ mind was the effect of the SSU on their cohort ranking rather than working well within the team and this reflected in the behaviours displayed by group members such as always wanting to go the extra mile’.

Finally, some indicated that teamwork was *nice* to know rather than *need* to know, as this student demonstrated when asked whether making time for teamwork was important.

Second interview: ‘I don’t think it’s essential to make time I think just being part of a team you just pick it up as you go along… just making time to chat people say at work having a tea break, chatting in an informal situation, getting to know them better then that helps the team work’.

## Discussion

### Importance and meaning of teamwork to students

Our students recognised that teamwork is crucial for effective health care and some also talked about teamwork in sport and home life. This is consistent with the literature, which generally agrees that students in later years of study know that teamwork is important for successful patient care [25, 26]. After the module, our students gave more in‐depth and personal responses on the importance of teamwork, bringing in examples they had seen during the year. This is reassuring from the perspective of the educator, although knowing that teamwork is important is not synonymous with accepting that bespoke teamwork education is necessary.

Before the module, our students had a superficial understanding of what made good and poor teamwork, not usually referring to specific behaviours. They identified good communication, shared goals and clear roles in teams as being important. The answers remained similar after the module, although the second responses were more detailed, more tangible and sometimes included references to their experience of the module, with more of them picking up ideas around identifying individuals’ strengths to improve the effectiveness of teamwork.

Students’ definitions of good and poor teamwork often invoked the repetition of a learned definition rather than deep understanding, perhaps reflective of the emphasis on recalling knowledge over original thought. Many interviewees referred to the need for a strong leader, but simultaneously that strict hierarchy was a barrier to effective team communication. When probed, students found it difficult to reconcile these paradoxes, something that may be resolved through further experience of team dynamics and teamwork training.

### Student insight into skill development

When asked in the first interview about the areas of teamwork they thought they were good at and where they could improve, the students gave general answers without clear ideas as to *how* they might improve. The difference in the second interview was quite marked, with students demonstrating insight into how they work best in teams, what they might do in future as a result of this experience and reflecting on how they have developed, including mentioning the importance of the reflective process.

We believe that the inclusion of reflective tasks has increased student insight into their development through the module, consistent with others who have also successfully used structured reflection in skill education [1, 31].

### Transferability of teamwork

Before the module, students were aware of the importance of teamwork in a clinical setting. After the module, students made more personal, in‐depth comments about teamwork in different contexts. It was more about them and their future careers rather than generic statements about health care. They also realised that, although teamwork is key to success in sport and in health care, there are subtle differences, with the example quoted referring to differences in communication styles.

Our use of authentic clinical placements has, in line with others’ findings, helped students develop their understanding of the transferability of teamwork to various contexts [17, 29, 30]. Our students have also demonstrated more detailed, personal descriptions of teamwork in different settings following the module. The value of placements (especially when combined with reflection) is true outside of clinical education too, with others reporting that authentic placements where students are immersed in a real workplace and given responsibility can catalyse the development of critical transferable skills across disciplines [1, 43, 44].

### Peer assessment in teamwork

We felt it was important to include some aspect of peer assessment in this module to increase the authenticity of the task and align it with the workplace, where our graduates will need to be skilled at giving and receiving appraisals and giving feedback to future medical students they will teach [45]. Indeed, feedback literacy (the ability to fully engage with and contribute to the process of feedback to support professional development) is an important transferable skill in all disciplines, not just medicine [46]. There are important challenges to consider when introducing peer assessment, and our students highlighted some of them in the interviews.

Our students recognised that peer assessment could be necessary and beneficial. Some also mentioned that they had already done other peer assessments elsewhere in the BMBS programme. However, when it comes to summatively assessing, or being summatively assessed by their peers, they are much warier of peer assessment. Some mentioned the impact of friendship groups, either that the ‘popular’ students would score highest or that it would be difficult to give an objective grade to their friends. They were also concerned about whether they had the necessary skills to carry out objective peer assessments. Some students discussed the propensity for individuals to exhibit behaviour in an inauthentic, ‘fake’ manner in order to meet the assessment requirements. There seems to be some appetite from students, at least in theory, to include peer assessment in this kind of module, but it is challenging to make it work well. Perhaps we need to emphasise (both to students and educators) that feedback literacy is a key competency within teamwork and that in order to succeed in a team, one must be able to give and respond to feedback in a constructive and professional manner. It may well be that our students need more training to be comfortable with this. Others have also found it challenging to implement effective peer assessment, and whilst we continue to examine students on their individual knowledge, and rank them accordingly, competition between students will continue to seriously undermine teamwork and peer assessment [22, 36].

### Student resistance to teamwork education

Whilst our interviews did not indicate overall dissatisfaction with the teamwork module, there were various comments that indicated some resistance from students. One theme that emerged was that students had already ‘done’ teamwork. They indicated that, as a result of other educational activities and life experience, they could already work in teams and/or that they just naturally acquired these skills as they went through life. This resonates with others who have challenged the perception held by many educators, that students just ‘pick up’ teamwork [18, 19] and that, more broadly, students are resistant to transferable skill education, and moving away from discipline‐specific content [47]. Our students did, however, appreciate the opportunity to practise their teamwork skills.

We also noted resistance related to the new module design, with comments made about the artificial or superficial nature of the activities and assessments, and general anxiety about the new module. We have attempted to strike a balance between letting our student teams develop organically and also allowing observation of real teams, whilst assessing their skills. Many others have also struggled to create appropriate teamwork assessments [4, 16, 23, 36, 48]. Students do value teamwork and rate it as important, even if their experience of specific modules is not entirely positive. We also need to remain aware that we, as educators, have different perspectives than our students and that students might not immediately recognise the value or relevance of transferable skill education [24, 47].

There is something fundamental in the way that we teach, assess and rank students, from primary education onwards that undermines the kind of education that rewards team success. Our students and others have specifically commented on the competition between peers in medical school [22]. In this study, our students were very frank about how important their rankings are, and that this creates conflict when they are being assessed on teamwork skills or team output. Success in university education is generally measured through individual students’ knowledge and understanding. Apart from specific clinical competencies, skills are rarely included in high‐stakes assessments and the system is not conducive to our attempts to develop and assess transferable skills. This is reflected in our students’ comments in this project. They see teamwork as *nice* to know rather than need to know. One student commented that making time for teamwork development was not essential and that they could just pick it up as they went, which is possibly symptomatic of the common and almost derogatory ‘soft‐skills’ epithet used to refer to these vital competencies. So despite the fact that our students emphatically and unanimously agreed that teamwork was vital to successful health care, they did not entirely back our method of providing teamwork education in their curriculum. When it comes to the crunch, perhaps our students prioritise knowledge over skills, because that is what they are tested on the most.

## Concluding remarks

Here, we have presented a qualitative evaluation of a novel teamwork module, designed to develop and assess medical students’ teamwork skills. Our module represents only one approach to promoting the development of teamwork. We hope that others will continue to develop alternative strategies and that we can share experiences to allow the teaching and assessment of transferable skills to evolve and take their rightful place alongside knowledge and clinical skills.

Our data suggest that this module has helped students travel a little way along their teamwork journey. They show subtle changes in some domains, demonstrating increased awareness of their own development, more reflection on their own teamwork skills and a more personal view of teamwork. It would be unrealistic to expect large measurable improvements in teamwork skills over a short timescale. Still, we hope that this module will sow seeds that bear fruit later on, as suggested by one of our students who thought the benefit of the module may be further revealed in future as they now have a better understanding of teamwork.

We are not alone in failing to demonstrate a marked increase in understanding aspects of teamwork through a single module [16, 49]. This is a complex challenge, arguably undermined by an education system that tests individual knowledge, and ranking that encourages competitiveness over cooperation. Teamwork in particular is troublesome to develop and assess, not only because is it a fairly intangible skill, compared with specific, factual, knowledge, but because it relies on the success of a group rather than an individual, thus creating conflict and anxiety in the highly competitive environment of a medical school.

Future work is needed to increase and improve our provision of transferable skill training across HE. We would like to further explore student perspectives on teamwork and how we can work constructively with our students to help them take ownership of their own skill development. Here, we have discussed one module, delivered during only 1 year of the medical programme. We would like to look at how spiralling of transferable skill training (year‐on‐year, for example) could facilitate development of these skills, and whether encountering them from day one of a degree programme might foster a different attitude towards transferable skills.

It is bold to base high‐stakes assessments on transferable skills, but we need to do it in order to prepare our graduates for the world. The importance of teamwork and other transferable skills cannot be underestimated. They are crucial to employability, success and enjoyment of work. More broadly, in health care and science, we need employees who are able to work well together for the best outcomes and to optimise well‐being in the workplace. Teaching and assessing this vital skill is challenging but surely not insurmountable.

## Conflict of interest

The authors have no conflict of interest to declare.

## Author contributions

HW, SB, TC, RJ and DZ conceived and designed the project. HW, RJ, DZ and SB designed the module evaluated here. HW was the principal investigator and wrote the manuscript, with contributions from all other authors. M‐KD, MP, JS, GB, TC, RJ and DZ conducted the interviews and transcriptions. All authors contributed to the thematic analysis of the data.

## Supporting information


**Appendix S1**. Questions for interview 1.

## Data Availability

The data that support the findings of this study are available from the corresponding author [helen.watson@plymouth.ac.uk] upon reasonable request.
