# Supplementary material for: ‘Everyone is trying to outcompete each other’: a qualitative study of medical student attitudes to a novel peer‐assessed undergraduate teamwork module
Source: FEBS Open Bio. 2022 Mar 23;12(5):900–12. doi: 10.1002/2211-5463.13395 (PMC9063444; doi:10.1002/2211-5463.13395)
Supplement: Supplementary file 1 — Appendix S1. Questions for interview 1. [file FEB4-12-900-s001.docx]

# Appendix

## Appendix 1

### Questions for interview 1

- How important is teamwork to you? Why?
- What do you think makes good teamwork?
- What do you think are barriers to good teamwork?
- What areas of teamwork do you think you’re good at?
- What areas of teamwork do you think you could be better at?
- What would you expect from a module that assesses teamwork?

### Questions for interview 2

- How important is teamwork to you? Why?
- What do you think makes good teamwork?
- What do you think are barriers to good teamwork?
- What areas of teamwork do you think you’re good at?
- What areas of teamwork do you think you could be better at?
- How do you feel about the teamwork SSU? Has your opinion on it changed since the start of the year?
- Do you think your understanding of teamworking has changed during the year? How do you feel this SSU has helped you develop your teamworking skills?
- What do you think about being assessed by your peers?
